# An adaptive variant of *TRIB2*, rs1057001, is associated with higher expression levels of thermogenic genes in human subcutaneous and visceral adipose tissues

**DOI:** 10.1186/s40101-017-0132-z

**Published:** 2017-02-17

**Authors:** Kazuhiro Nakayama, Sadahiko Iwamoto

**Affiliations:** Division of Human Genetics, Center for Molecular Medicine, Jichi Medical University, 3311-1 Yakushiji, Shimotsuke, 329-0498 Japan

**Keywords:** *TRIB2*, SNP, Thermogenesis, Obesity

## Abstract

**Background:**

An obesity-related single-nucleotide polymorphism (SNP) of the Tribbles pseudokinase 2 gene (*TRIB2*) was shown to have underwent adaptive evolution in the last glacial period, suggesting a selective advantage of this SNP in human populations in cold environments. In order to verify this hypothesis, the effect of the *TRIB2* SNP on the expression of genes involved in adaptive thermogenesis was tested using messenger RNAs prepared from adipose tissues of Japanese adults.

**Methods:**

Complementary DNA was prepared from subcutaneous adipose tissues (SAT) and visceral adipose tissues (VAT) obtained from 48 Japanese adults. Transcript levels of 15 selected genes, including five genes that are upregulated in development of thermogenic adipocytes, were measured by using real-time polymerase chain reaction. Differences in transcript levels between the *TRIB2* SNP genotype groups (AA genotype versus AT + TT genotype) were assessed using *t* test.

**Results:**

Of the five thermogenic genes, *DIO2*, *CIDEA*, *PPARGC1A*, and *PRDM16* showed significantly higher transcript levels in SAT of individuals with the AA genotype relative to those with the AT + TT genotype (*P* < 0.05). However, only 2 out of the 10 non-thermogenic genes exhibited differences in transcript levels according to genotype. Additionally, *in silico* prediction indicated that this SNP likely affects the expression of nearby genes including *TRIB2*.

**Conclusion:**

The higher expression levels of thermogenic genes in individuals homozygous for the adaptive variant of *TRIB2* SNP suggest a greater propensity for induction of thermogenesis in adipose tissues in cold environments.

## Background

The primary cause of obesity is an imbalance between caloric intake and energy expenditure. Susceptibility to obesity in present human populations is thought to be shaped by past genetic adaptation to famine [1, 2]. Additionally, lower ambient temperatures may exert selective pressure on genetic variations that influence susceptibility to obesity, as cold-induced thermogenesis substantially increases energy expenditure [3]. It may be hypothesized that genotypes linked to higher thermogenic capacity and leaner phenotypes were adaptive in archaic human populations during glacial periods [4]. A single-nucleotide polymorphism (SNP) in the uncoupling protein 1 gene, an essential gene for thermogenesis in brown adipocytes, has been shown to support the role of adaptation to cold climates in shaping the susceptibility to obesity [5, 6].

A previous study reported that a functional SNP in the 3′ untranslated region of the tribbles pseudokinase 2 gene (*TRIB2*) strongly influences visceral fat accumulation in Japanese adults. The obesity-resistance-associated allele of *TRIB2* underwent positive natural selection in East Asians during the last glacial maximum, suggesting that this *TRIB2* variant links past adaptation to present resistance to obesity [7]. *TRIB2* suppresses the differentiation of adipocytes by promoting the degradation of the CCAAT/enhancer binding protein beta (CEBPB), a transcription factor that acts during early stages of brown adipocyte development [8, 9]. Human brown adipocytes are thought to be present only during early life in humans; however, recent studies have indicated the existence of active brown adipocytes in adult humans [10]. Moreover, it has been shown that energy-storing white adipocytes (or/and their progenitor cells) may be transformed into thermogenic beige/brite adipocytes via a series of stimuli; these thermogenic adipocytes resemble brown adipocytes but possess distinctive developmental lineages [11]. The adaptive *TRIB2* allele, which is associated with the reduction of visceral fat, may contribute to resistance to cold environments by increasing the activity of these thermogenic adipocytes. In the present study, we examined the effects of the previously reported *TRIB2* SNP on the expression of genes involved in thermogenesis in the adipose tissues of Japanese adults.

## Methods

Subcutaneous adipose tissue (SAT) and visceral adipose tissue (VAT) samples were obtained from 48 Japanese adults. These participants were enrolled from among patients admitted to the Jichi Medical University Hospital for gastrointestinal surgery or gynecological surgery. All participants provided written informed consent. SAT was collected from the abdominal skin incision and VAT from the extirpated organ, omentum, or mesenterium during surgical procedures. Complementary DNA (cDNA) and genomic DNA were prepared from adipose tissue specimens. Details of participants and samples were described in the previous paper [12]. The *TRIB2* SNP (dbSNP: rs1057001) was genotyped using TaqMan Genotyping Assays and ABI PRISM 7900HT [7]. Transcription levels of selected genes were measured using TaqMan Gene Expression Assays or RT-PCR with SYBR Green on an ABI PRISM 7900HT. The list of target genes is shown in Table 1. Transcript levels of the target genes were normalized to that of the actin beta gene (measured by the TaqMan method) using the delta Ct method. Statistical tests were performed using SPSS version 23. Epigenetic modification patterns near rs1057001 and tightly linked variants (*r* ^2^ > 0.8 in the 1000 Genome Project East Asians) were retrieved using Haploreg4.1 [13]. The design of the present study was approved by the ethical committee of Jichi Medical University.

**Table 1:** Tested genes

Symbol	Gene name	Function	Assay type and nucleotide sequences of primers^a^
*CIDEA*	Cell death-inducing DFFA-like effector A	Development of brown/beige adipocytes	5′-CAGCTCGCCCTTTCCGGGTC 5′-CGAGGGCATCCAGAGTCTTGCT
*DIO2*	Iodothyronine deiodinase 2	Thermogenesis	5′-AGAGGGACTGCGCTGCGTCT 5′-TGCACCACACTGGAATTGGGGG
*PDRM16*	PR/SET domain 16	Development of brown/beige adipocytes	5′-GAACCAGGCATATGCAATGATGCTG 5′-CCAGCCCGTCAGAGGTGGTTG
*PPARGC1A*	PPARG coactivator 1 alpha	Development of brown/beige adipocytes	5′-AGACACCGCACGCACCGAAAT 5′-AGCTGTCATACCTGGGCCGACG
*UCP1*	Uncoupling protein 1	Thermogenesis	5′-CGGCCTCTACGACACGGTCCA 5′-ACGACCTCTGTGGGTTGCCCAA
*ACC*	Acetyl-CoA carboxylase	Fatty acid synthesis	TaqMan
*DGAT1*	Diacylglycerol O-acyltransferase 1	Triglyceride synthesis	5′-GCCCCCAACAAGGACGGAGAC 5′-CCACACACCAGTTCAGGATGCCA
*FASN*	Fatty acid synthase	Fatty acid synthesis	TaqMan
*SCD1*	Stearoyl-CoA desaturase 1	Fatty acid synthesis	TaqMan
*SREBP1*	Sterol regulatory element-binding protein1	Master regulator of fatty acid synthesis	TaqMan
*ADIPOQ*	Adiponectin	Adipocytokine	TaqMan
*FABP4*	Fatty acid-binding protein 4	Incorporation of fatty acids	5′-TGGGGGTGTCCTGGTACATGTGC 5′-ACGCCTTTCATGACGCATTCCACC
*ADRB3*	Adrenoceptor beta 3	Lipolysis	TaqMan
*GLUT4*	Facilitated glucose transporter, member 4	Incorporation of glucose	5′-TTGGCCCTGGCCCCATTCCT 5′-GCCCCATAGCCTCCGCAACA
*LEP*	Leptin	Adipocytokine	5′-TAGGAATCGCAGCGCCAGCGG 5′-ATCCGCACAGGGTTCCCCAATG

^a^Primer sequences are shown for genes quantified by RT-PCR with SYBR Green

## Results and discussion

The genotypes of rs1057001 in participants were AA = 26, AT = 20, and TT = 2. The proportion of the three genotypes was in Hardy-Weinberg equilibrium status (*P* = 0.421, chi square test, degrees of freedom = 1). Individuals with TT genotype were infrequent and thus combined with individuals with AT genotype for further statistical analyses. Genotype groups did not show differences in terms of the proportion of males and females (*P* = 0.066, Fisher’s exact test), age (*P* = 0.253, *t* test), and body mass index (*P* = 0.960, *t* test).

The transcript levels of tested genes are shown in Table 2. We tested *CIDEA*, *DIO2*, *PPARGC1A*, *PRDM16*, and *UCP1*, which were previously linked to brown adipose tissue activity as well as induction of beige/brite adipocytes in mice and humans [14–18]. The *UCP1* transcript was undetectable in the majority of the adipose tissue samples. Transcripts of the other four genes were present at higher levels in individuals with the AA genotype than in those with the AT + TT genotype (*P* < 0.05, *t* test). The level of *PPARGC1A* expression in SAT was significant, at 5% after the Bonferroni correction (numbers of successfully measured gene = 14). The genotypic differences in the transcript levels of thermogenic genes were more notable in SAT, in which induction of beige/brite adipocytes was observed in mice and humans [11]. Furthermore, *CIDEA* and *PRDM16* showed higher expression levels in SAT than in VAT (*P* < 0.05, after the Bonferroni correction was applied), supporting the greater prepotency of SAT for thermogenesis. We additionally tested the effect of *TRIB2* genotypes on the expression of genes for *de novo* lipogenesis in white adipocytes (*ACC*, *DGAT1*, *FASN*, *SCD1*, *SREBP1*) and genes commonly expressed in white and brown adipocytes (*ADIPOQ*, *ADRB3*, *FABP4*, *GLUT4*, and *LEP*). Of the 10 genes, only *ADIPOQ* and *GLUT4* in SAT showed significant differences in expression between the genotype groups (*P* < 0.05, *t* test).

**Table 2:** Transcription levels of the tested genes in the *TRIB2* genotype groups

Gene symbol	Mean (SD) transcription level in subcutaneous adipose tissues		Ratio	*P*	Mean (SD) transcription level in visceral adipose tissues		Ratio	*P*
	AA	AT + TT			AA	AT + TT		
*CIDEA*	0.4474 (0.1986)	0.2999 (0.2094)	1.45	0.016	0.2962 (0.2511)	0.1897 (0.1572)	1.55	0.095
*DIO2*	0.0012 (0.0017)	0.0005 (0.0003)	2.51	0.036	0.0012 (0.0009)	0.0006 (0.0007)	2.00	0.015
*PPARGC1A*	0.0023 (0.0015)	0.0012 (0.0005)	1.80	0.002	0.0017 (0.0009)	0.0012 (0.0009)	1.51	0.034
*PRDM16*	0.0074 (0.0058)	0.0041 (0.0033)	1.73	0.021	0.0028 (0.0022)	0.0018 (0.0014)	1.58	0.084
*ACC*	0.0054 (0.0038)	0.0082 (0.0061)	0.65	0.065	0.0045 (0.003)	0.006 (0.0034)	0.75	0.112
*DGAT1*	0.0405 (0.024)	0.044 (0.0227)	0.96	0.61	0.0935 (0.0371)	0.1052 (0.0652)	0.88	0.440
*FASN*	0.2635 (0.1727)	0.4259 (0.3704)	0.60	0.069	0.2006 (0.1452)	0.2739 (0.2178)	0.72	0.172
*SCD1*	0.8676 (0.8784)	1.3172 (1.7992)	0.64	0.266	0.7727 (0.7134)	0.8298 (1.0251)	0.72	0.822
*SREBP1*	0.0238 (0.0126)	0.0366 (0.0268)	0.64	0.050	0.0255 (0.0345)	0.0213 (0.0138)	1.19	0.577
*ADIPOQ*	0.5757 (0.2606)	0.8252 (0.4946)	0.69	0.041	0.4176 (0.3337)	0.4953 (0.2959)	0.85	0.402
*ADRB3*	0.002 (0.0021)	0.0017 (0.0023)	1.13	0.667	0.0017 (0.0018)	0.0021 (0.0027)	0.80	0.590
*FABP4*	1.683 (0.84)	1.2587 (0.6681)	1.31	0.068	0.7458 (0.4979)	0.718 (0.4251)	1.09	0.839
*GLUT4*	0.0463 (0.0462)	0.0241 (0.0192)	1.86	0.035	0.0246 (0.016)	0.0175 (0.0141)	1.45	0.114
*LEP*	0.0803 (0.0847)	0.0653 (0.0539)	1.24	0.477	0.0189 (0.0151)	0.0222 (0.0321)	1.02	0.643

AA and AT + TT indicate *TRIB2* genotypes. “Ratio” indicates mean expression levels of AA where the mean expression levels of AT + TT were set to be 1. Data for *UCP1* are not shown as *UCP1* transcripts were not detectable in the adipose tissue samples. SD indicates standard deviation

**Fig. 1:**
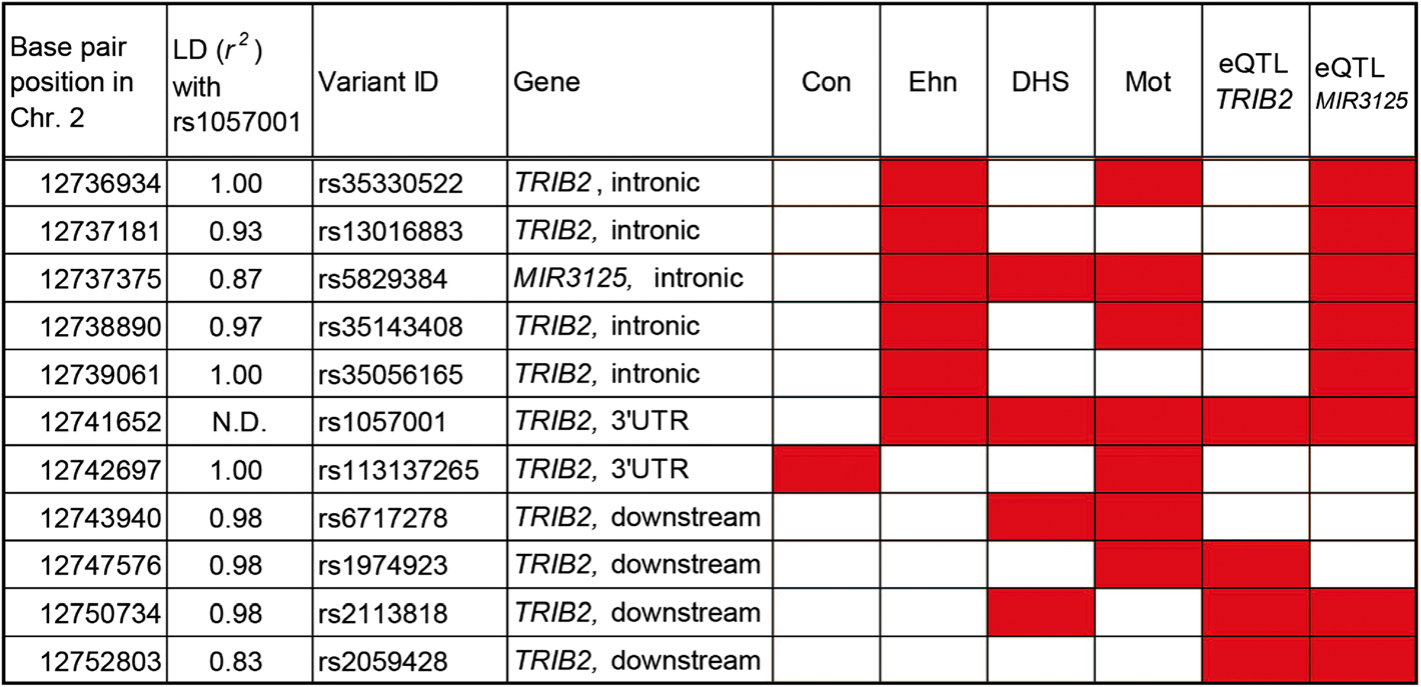
Functional annotation of the short nucleotide sequence variants in strong linkage disequilibrium (LD) with rs1057001. *Red color* indicates SNPs located in an evolutionarily conserved site (Con), in enhancer histone marks in adipocytes (Ehn), in DNase hypersensitive site (DHS), and in protein-binding motif sequences (Mot). Nucleotide sequence variants associated with expression levels of nearby genes (*TRIB2* and/or *MIR3125*) in previous studies are also indicated in *red*. The functional annotation of the nucleotide sequence variants was performed using Haploreg4.1

The previous study showed that the A allele of rs1057001 is linked to lower transcription levels of *TRIB2* in adipose tissues [7]. We additionally investigated epigenetic modifications of rs1057001 and short nucleotide sequence variants with tight linkage disequilibrium status with rs1057001 (Fig. 1). Several of these variants were found in genome sequences with signatures of gene regulatory elements, including histone enhancer marks, DNase hypersensitive sites, and alteration of protein-binding motifs. These variants are considered to coordinately alter expression levels of *TRIB2* in various tissues. The transcription level of a microRNA-encoding gene (*MIR3125*) as well as of *TRIB2* showed significant correlations with genotypes of rs1057001 or the tightly linked variants. Although the physiological function of *MIR3125* is still unknown, this gene is thought to contribute to the genotype-related effects of the *TRIB2* variant of the thermogenic genes.

Higher expression levels of genes involved in the differentiation and function of brown and beige/brite adipocytes may indicate a greater propensity for thermogenesis in individuals with the AA genotype in response to cold stimuli. This observation may support the hypothesis that the A allele contributed to cold adaptation in ancestors of East Asians in the last glacial period [7]. The suppression of *Trib2* has been shown to reduce the degradation of CEBPB, a key transcription factor involved in brown adipocyte differentiation in murine models [8, 9]. This suggests that the AA genotype, which is linked with suppressed expression of *TRIB2*, is associated with more potent differentiation of thermogenic adipocytes. Transcription levels of *UCP1*, an important component of the thermogenic machinery of brown and beige/brite adipocytes, were very low in the present adipose tissue samples. *UCP1* expression is known to occur at very low levels in non-stimulated white adipose tissues in mice and humans [18, 19]. The present adipose tissue samples, which were obtained from patients who were not subject to cold simulation, did not exhibit high levels of *UCP1* expression.

The present study has several potential limitations, which should be addressed in future work; (1) the observed gene expression pattern may be confounded by non-adipocyte cell populations, mainly stromal vascular cells, in the adipose tissues. (2) It is unclear whether the abdominal SAT mirrors the white adipose tissues in the supraclavicular region, which represents the main site of expression of thermogenic genes in human beige/brite adipocytes [19]. (3) The molecular mechanisms underlying the modulation of thermogenic gene expression by *TRIB2* remain unknown.

In conclusion, the present study provides evidence for the role of *TRIB2* in energy expenditure in adipose tissues. These findings are believed to explain the previously proposed positive natural selection for the *TRIB2* cold-adaptive variant during the last glacial period.

## Abbreviations

ACC: Acetyl-CoA carboxylase
ADIPOQ: Adiponectin
ADRB3: Adrenoceptor beta 3
cDNA: Complementary DNA
CEBPB CCAAT: Enhancer binding proteinbeta
CIDEA: Cell death-inducing DFFA-like effector A
DGAT1: Diacylglycerol O-acyltransferase 1
DIO2: Iodothyronine deiodinase 2
DNA: Deoxyribonucleic acid
FABP4: Fatty acid-binding protein 4
FASN: Fatty acid synthase
GLUT4: Facilitated glucose transporter, member 4
LD: Linkage disequilibrium
LEP: Leptin
PCR: Polymerase chain reaction
PDRM16: PR/SET domain 16
PPARGC1A: PPARG coactivator 1 alpha
RNA: Ribonucleic acid
RT-PCR: Real-time PCR
SAT: Subcutaneous adipose tissues
SCD1: Stearoyl-CoA desaturase 1
SNP: Single-nucleotide polymorphism
SREBP1: Sterol regulatory element binding protein1
TRIB2: Tribbles pseudokinase 2
UCP1: Uncoupling protein 1
VAT: Visceral adipose tissues
